# An Overview of the Biological Complexity of Vitiligo

**DOI:** 10.1155/omcl/3193670

**Published:** 2024-12-19

**Authors:** Paola Matarrese, Rossella Puglisi, Gianfranco Mattia, Tonia Samela, Damiano Abeni, Walter Malorni

**Affiliations:** ^1^Center for Gender-Specific Medicine, Istituto Superiore di Sanità (ISS), Rome, Italy; ^2^Clinical Psychology Unit, Istituto Dermopatico dell'Immacolata (IDI) IRCCS, Rome, Italy; ^3^Clinical Epidemiology Unit, Istituto Dermopatico dell'Immacolata (IDI) IRCCS, Rome, Italy; ^4^Center for Global Health, Università Cattolica del Sacro Cuore (UCSC), Rome, Italy

**Keywords:** adrenergic receptors, biological factors, epidermal fibroblasts, gender, keratinocytes, melanocytes, MiRNA, mitochondria, oxidative stress, sex, sex hormones, skin pigmentation

## Abstract

Vitiligo is a skin disease that affects all ethnicities and genders and is characterized by the loss of pigment essentially due to the selective loss of melanocytes. Although it is generally considered a systemic disease associated with polymorphisms in genes involved in the immune response, vitiligo is also considered an oxidative imbalance-associated disease. It represents a multifactorial pathology in which some genetic predisposition and epigenetic factors coupled with some critical biochemical and molecular pathways could play a pivotal role. The aim of this work was thus to review some of the fine cellular mechanisms involved in the etiopathogenesis of vitiligo, mainly focusing on the nonimmunological ones, extensively highlighted elsewhere. We took into consideration, in addition to oxidative stress, both the cause and the hallmark of the pathology, some less investigated aspects such as the role of epigenetic factors, e.g., microRNAs, of receptors of catecholamines, and the more recently recognized role of the mitochondria. Sex differences associated with vitiligo have also been investigated starting from sex hormones and the receptors through which they exert their influence. From literature analysis, a picture seems to emerge in which vitiligo can be considered not just a melanocyte-affecting disease but a systemic pathology that compromises the homeostasis of a complex tissue such as the skin, in which different cell types reside playing multifaceted physiological roles for the entire organism. The exact sequence of cellular and subcellular events associated with vitiligo is still a matter of debate. However, the knowledge of the individual biological factors implicated in vitiligo could help physicians to highlight useful innovative markers of progression and provide, in the long run, new targets for more tailored treatments based on individual manifestations of the disease.

## 1. Introduction

Vitiligo is a skin disease clinically characterized by loss of pigment that can affect areas of skin of varying extent, from a few millimeters to several centimeters. It is an acquired pathology in which the development of white patches is linked to the selective loss of melanocytes. The prevalence of vitiligo is 0.5%–2.0% in the general population worldwide; in Europe and the United States, it is around 1% [1, 2]. A very recent systematic review, analyzing data from 90 cross-sectional and cohort studies, reports an overall global prevalence of vitiligo of 0.36%, affecting ~28.5 million people worldwide, including adults and children [3]. Surprisingly, in certain countries, such as Mexico or India, it can reach up to 8% of the population [4]. It has been hypothesized that there may be a link with chemical exposure [5].

Vitiligo is an immune-mediated systemic disease associated with polymorphisms in genes involved in the immune response. The autoimmune nature of vitiligo has been suggested by several studies demonstrating that both the adaptive immune system, through cytotoxic CD8+ T lymphocytes and specific antibodies against melanocytes, and the innate immune system can exert a pathogenetic role [6, 7].

Vitiligo has a strong hereditary component. Loci and allelic variants of some genes implicated in the development of vitiligo have been identified. Among these are genes responsible for the expression of proteins involved in immune regulation, apoptosis, and melanocyte functions [8]. However, environmental factors are necessary for the clinical manifestation of the disease [8]. Among these, sunburn, hormones, and biological, mechanical, and chemical stressors have been identified. Among chemicals, it has been hypothesized that phenolic and catechol derivatives could play an important role [5]. Since the melanocytes of patients with vitiligo show high susceptibility to reactive oxygen species (ROS)-induced injury and death, it has been hypothesized that oxidative stress could play a major role [9]. ROS induce an immunogenic cell death type, which, facilitating the formation of autoantigens, would further increase the loss of melanocytes by igniting an autoimmune reaction [10]. It is interesting to note, however, that the presence of autoantibodies against melanocytes in serum do not necessarily correlate with disease activity [11]. This seems to indicate once again that the immune event is necessary but not sufficient to activate vitiligo.

Vitiligo occurs before the age of 20 in approximately half of patients [12]. Late onset is often associated with other immune-mediated pathologies such as thyroid dysfunction, rheumatoid arthritis, diabetes mellitus, and alopecia areata [12]. With regard to the incidence, it seems to be higher and with greater social impact in subjects with pigmented skin, while the male/female ratio in vitiligo is still under debate. In fact, some authors reported that vitiligo was more common in men and others in women [13, 14], whereas other investigators found no significant differences between the two sexes [15, 16]. These discrepancies could be due to the different methodologies used, e.g., to a different stratification of the subjects taken into consideration or to different risk factors considered. However, since most of these studies involve a limited number of patients and healthy controls (a few dozen to a few hundred), the results may not be generalizable. A recent systematic review limited to Africa, the Middle East, and Latin America found no statistically significant gender difference in the prevalence of vitiligo, at least in these countries [15]. Similarly, a recent article based on a cohort study and a cross-sectional study including children, adolescents, and adults diagnosed with vitiligo in the United States from 2015 to 2019 considering over 2 million subjects clearly indicated that there are no significant differences in incidence and prevalence between males and females [16]. The prevalence of the pathology in the female gender that has emerged from some studies is most likely linked to the greater demand for medical care by women for whom vitiligo represents an impacting esthetic problem. In fact, based on numerous published works, there are significant gender differences in the manifestations of psychological suffering associated with vitiligo [17, 18]. However, this matter is beyond the scope of this review, which takes into consideration only the biological aspects of vitiligo.

**Figure 1:**
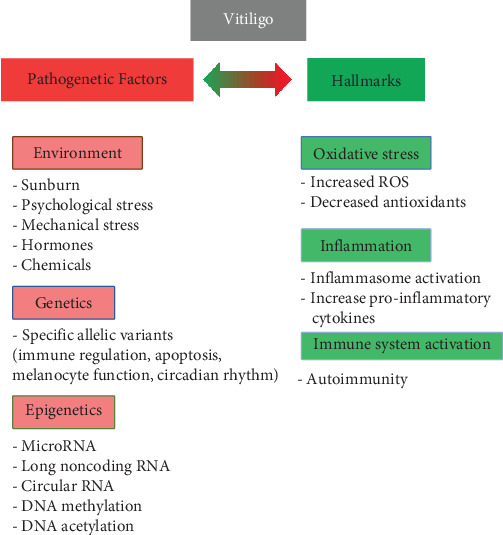
The main pathogenetic factors and hallmarks that underline how vitiligo is a multifactorial pathology. These determinants can have a different weight in different clinical cases. ROS, reactive oxygen species.

From the etiopathogenetic point of view, all authors now agree in considering vitiligo a multifactorial pathology (Figure 1). Although, as with most diseases, a certain genetic predisposition to vitiligo has been observed, epigenetic (microRNA, DNA methylation, etc.) and environmental factors have also been hypothesized of relevance in the onset, progression, clinical manifestations, and response to therapy of this disease [19–21]. Numerous anomalies have been found in melanocytes isolated from subjects with vitiligo compared to healthy individuals: low proliferative index, dilation of the endoplasmic reticulum, altered morphology and aggregation of melanosomes [22], altered lipid composition [23], decreased oxygen consumption, aberrant mitochondrial complexes I, II, and IV, and respiratory responses [24] contributing to vulnerability of melanocytes to different types of cell death [25].

Based on the above results and hypotheses, multiple molecular mechanisms could contribute to the loss of melanocytes observed in vitiligo while the contribution of each individual aspect to the development of the disease has yet to be ascertained. In addition, it is not known whether segmental (i.e., when there is a rapid color loss on one side of the body) and nonsegmental (i.e., when patches tend to appear on both sides of the body like both knees or both hands) vitiligo involve the same pathogenetic mechanisms.

Over the years, numerous theories have been proposed to explain the pathogenesis of vitiligo, each of which, however, has the limit of privileging some mechanisms over others depending on the biological areas investigated by the individual research groups [26, 27]. Although some researchers have attempted to synthesize these theories into a “convergent theory” [28], the *primum movens*, if it exists at all, has not yet been identified. It cannot be excluded that the true culprit may be different in different clinical cases.

This paper aimed to review some of the biological mechanisms involved in the etiopathogenesis of vitiligo, focusing mainly on the nonimmunological ones, as the immunological ones have already been extensively investigated [20, 29, 30]. In particular, we will focus on papers published in the last 8 years without failing to mention less recent ones that appear particularly important in the field of study of vitiligo.

## 2. The Complex Role of Oxidative Stress

During melanogenesis, various ROS are generated, and this makes melanocytes particularly susceptible to oxidative stress [31]. Among the ROS produced during melanin synthesis, superoxide anion and hydrogen peroxide are the more relevant. To protect the cellular components of melanocytes from oxidative damage, melanin synthesis is compartmentalized within specialized organelles, melanosomes. Their integrity appears thus as an essential condition for melanocyte survival. In fact, the case of vitiligo seems to be paradigmatic since oxidative stress represents one of the main pathogenetic factors of this disease [32]. The excessive production of ROS, or their accumulation, interrupts the process of melanin synthesis and the circulation of lipids in melanocytes, inducing damage to the mitochondrial electron transport chain, which in turn induces an increase in ROS production, creating an evil loop which leads to oxidative stress and the consequent death of melanocytes [33]. Oxidative stress is also a proinflammatory factor that promotes the release of cytokines from keratinocytes with consequent migration of CD8+ T lymphocytes into the skin tissue. The release of cytokines by CD8+ T lymphocytes induces a further amplification of the immune response with consequent damage to melanocytes [34]. Accordingly, significantly increased levels of inflammatory cytokines, including IL-15, IL-17, and IL-1β, are found both in the perilesional dermis and peripheral blood of patients with vitiligo. Most of these interleukins enhance the cytotoxic functions of CD8+ T lymphocytes. The increase of IL-15 induces the production of IFN-γ-inducible cytokines, CXCL9 and CXCL10 [35], whereas IL-17 and IL-1β would act by activating T helper-17 cells, inducing mitochondrial dysfunction and consequent cellular damage [36]. Inhibition of this chemotactic axis could thus represent a promising therapeutic option. IFN-γ appears to be involved in the early onset of vitiligo. It has been observed that the increased level of IFN-γ activates, via JAK 1/2, the transcription of CXCL9 and CXCL10, necessary for recruiting cytotoxic T-lymphocytes, and induces an overexpression of intercellular adhesion molecule 1 (ICAM-1) on melanocytes, thus favoring their cytokine-mediated destruction [37, 38]. These immunological aspects, which we decided to exclude from this literature review, are well described elsewhere [20].

Regarding cell redox imbalance, nuclear factor erythroid 2-related factor 2 (NRF2) regulates the expression of antioxidant proteins protecting against cellular oxidative damage triggered by injury and inflammation. NRF2 is a transcription factor encoded by the NFE2L2 gene, which plays a pleiotropic role by regulating metabolism, inflammation, mitochondrial function, and immune response [39]. NRF2 translocates to the nucleus where it binds to the antioxidant response element (ARE) inducing the transcription of its numerous target genes, including antioxidants, detoxifying enzymes, and stress response proteins. Among these are the enzymes manganese superoxide dismutase (MnSOD), catalase, peroxidases (Prxs), glutathione peroxidases (GPxs), gamma cysteine ligase (GCL), heme oxygenase-1 (HO-1), and NADPH-ouinone-oxidoreductase 1 (NQO-1) [40]. NRF2 also reduces the oxidized cysteine residues of redox-active proteins and inhibits the NOD-, LRR-, and pyrin domain-containing protein 3 (NLRP3) inflammasome through activation of thioredoxin 1 (TXN1), thus playing the dual role as antioxidant and anti-inflammatory [41]. According with the crucial role of NRF2 in skin cell function under both physiological and pathological conditions, it were reported lower levels of NRF2 in melanocytes isolated from subjects affected by vitiligo compared to healthy individuals [42]. A reduction of the nuclear localization of NRF2, and consequently of its transcriptional activity, was also found in vitiligo melanocytes [42]. The alteration of the cytoplasm-nuclear translocation processes of NRF2 may therefore contribute to the dysfunction and death of melanocytes due to an insufficient antioxidant cell response [43].

Further studies have highlighted an imbalance of redox state also in peripheral blood of vitiligo subjects, in lesional epidermis, and in cultures of melanocytes isolated from the perilesional skin of vitiligo patients. As mentioned above, among the endogenous antioxidants found altered in individuals with vitiligo are the enzymes catalase (CAT), SOD, and GPx, and some vitamins such as C and E [44–46]. The presence of oxidative stress at a systemic level in patients with vitiligo would also be confirmed by the presence in the serum of lipid peroxidation products, such as malondialdehyde (MDA), and of oxidative DNA damage, such as 8-hydroxy-2-deoxyguanosine (8-OHdG) [47, 48]. However, although this central role of oxidative stress, no ROS-associated markers of clinical use for monitoring disease activity and/or response to therapy have been identified so far (Figure 1).

## 3. Not Just a Melanocyte-Affecting Disease

The cross talk between epidermal and dermal cells is the basis of the skin's functionality, both in physiological and pathological conditions. Vitiligo can be considered a pathology that compromises the homeostasis of a complex tissue such as the skin, which plays a likewise complex physiological role for the entire organism and in which different cell types reside [49]. The interaction among melanocytes and their cellular partners, keratinocytes and dermal fibroblasts, is fundamental for determining the color and correct functionality of the skin [50]. Thus, it is reasonable to assume that the alteration of the melanocyte–keratinocyte–fibroblast dialog could contribute to the development of vitiligo.

It is known that the adhesion molecule E-cadherin plays an important role in the functional connection between melanocytes and keratinocytes in the epidermis [51].

A deficit of the adhesive properties of melanocytes was observed in the lesional and nonlesional areas of the skin of subjects with vitiligo. In particular, the melanocytes of nonlesional areas showed a reduced expression of E-cadherin at the plasma membrane. The low levels of E-cadherin in melanocytes have been associated with their altered positioning in the suprabasal area and with an altered physical and biochemical interaction with the keratinocytes of the basal layer. In addition, the reduction of E-cadherin also makes melanocytes significantly more susceptible to chemical and mechanical stress perhaps due to functional disconnection with keratinocytes. It has been suggested that reduced expression of E-cadherin in melanocytes may precede the clinical manifestations of the disease [52].

Keratinocytes also represent the main source of aquaporin-3 (AQP3) in the skin [53]. AQP3 is a water and glycerol channel protein that, in addition to being involved in skin hydration, regulates the absorption of hydrogen peroxide [54] and participates in hetero- and homo-cellular contacts through a direct physical interaction with E-cadherin [55]. The downregulation of AQP3 observed in vitiligo keratinocytes of injured areas compromises their antioxidant responses and hydrogen peroxide absorption. The consequent increase in ROS concentrations in the surrounding environment could therefore contribute to the loss of nearby melanocytes [56]. Keratinocytes produce several cytokines and chemokines found involved in vitiligo [57]. Furthermore, keratinocytes also secrete numerous mediators impacting melanocytes such as endothelin 1 (ET-1), stem cell factor (SCF), basic fibroblastic growth factor (bFGF), and the granulocyte-monocyte colony stimulating factor (GM-CSF), which inducing proliferation and differentiation of melanocytes, and melanogenesis contribute to skin pigmentation [58].

Keratinocytes are also known to stimulate the synthesis of growth factors in fibroblasts to further stimulate their proliferation. On the other hand, in a proinflammatory microenvironment, such as that of vitiligo, keratinocytes can induce fibroblasts to differentiate into myofibroblasts [59].

Keratinocytes from subjects affected by vitiligo also show numerous morphological and ultrastructural anomalies, such as reduction of keratin filaments and desmosomes, vacuolization, and mitochondrial alterations [60]. At the biochemical level, a reduced amount of the protein catalase was observed, although no significant reduction was detectable at the mRNA level [61]. These alterations have been found in lesional, perilesional and nonlesional skin areas [22]. Furthermore, keratinocytes isolated from the lesional areas of the skin showed some phenotypic senescence traits, such as a reduced number of duplications *in vitro*, and a lower ability to support melanocytes growth *in vitro* compared to that of keratinocytes isolated from nonlesional areas [62]. According with this, in patients affected by vitiligo, following skin injuries the reepithelization process appears significantly delayed in the lesional areas compared to the nonlesioned ones [59], strongly suggesting an alteration in the relationships between keratinocytes and melanocytes, which can appear concomitantly with, precede, or follow the disappearance of the melanocytes. Similarly to melanocytes, alterations due to oxidative stress (i.e., increased production of ROS, and/or decrease in antioxidant capacity), increased apoptosis (i.e., activation of caspase 3), and mitochondrial dysfunction have also been described in perilesional keratinocytes. Furthermore, keratinocytes from depigmented skin areas showed reduced calcium uptake and lifespan, and increased expression of senescence markers [63]. In addition to the dysregulated production of numerous cytokines of pathogenetic value, the keratinocytes of the damaged and non-damaged skin areas present alterations in glutathione S-transferase (GST) activity, increase in glutathione peroxidase and SOD, and reduction of catalase and of some vitamins with antioxidant activity such as vitamin E [36]. The accumulation of MDA, and the reduction of polyunsaturated fatty acids, also demonstrated the presence of a lipoperoxidation phenomenon in vitiligo keratinocytes [64]. In this regard, it has been observed that exposing normal keratinocytes to sub-cytotoxic concentrations of 4-hydroxy-2-nonenal (HNE) induced alterations like those found in vitiligo keratinocytes [65]. Lipoperoxidation products such as HNE could therefore play a role in the pathogenesis of vitiligo by contributing to the dysfunctions of keratinocytes, as well as of melanocytes.

As far as the dermal fibroblasts is concerned, together with the extracellular matrix (ECM), they constitute a source of chemical and mechanical signals that connect the different skin cells. ECM participates directly, through the distribution and degradation of melanin, and indirectly, by stimulating keratinocytes to produce support factors in the growth and differentiation of melanocytes, to the skin pigmentation. Phenotypic characteristics typical of aging, such as enlargement and flattening of the cell body, and a substantial increase in actin stress fibers have been observed in fibroblasts from vitiligo subjects. In addition, a phenotypic switch with *de novo* expression of alpha smooth muscle actin (*α*-SMA) was also found in fibroblast of lesional areas [64]. Some soluble factors produced and secreted by fibroblasts have been observed to possess inhibitory activity on pigmentation; for instance, Dickkopf 1 (DKK1), highly expressed in physiologically hypo-pigmented skin areas (i.e., palmoplantar), and IL-6, an inflammatory cytokine found elevated in vitiligo with an inhibitory effect on tyrosinase activity [66].

**Figure 2:**
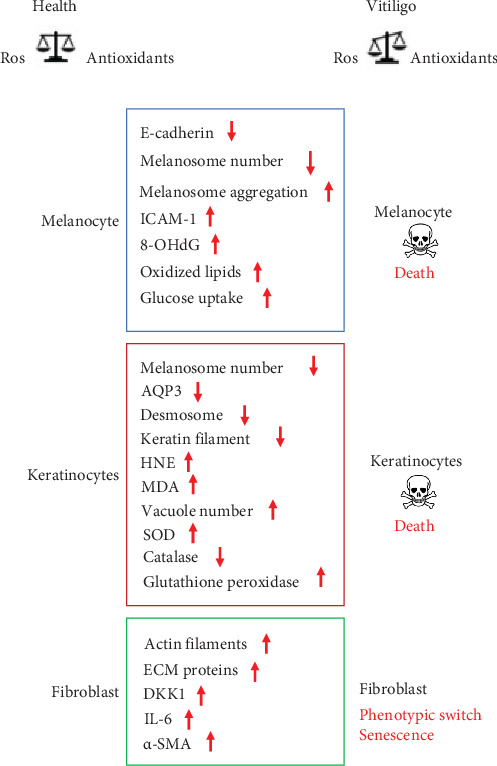
The main alterations observed in melanocytes, keratinocytes, and fibroblasts of patients affected by vitiligo in different phases of the pathology, indiscriminately in the lesional, perilesional, or distant areas from the lesions. AQP3, aquaporin-3; *α*-SMA; alpha smooth muscle actin; DKK1, Dickkopf 1; ECM, extracellular matrix; 8-OHdG, 8-hydroxy-2-deoxyguanosine; HNE, 4-hydroxy-2-nonenal; ICAM-1, intercellular adhesion molecule 1; MDA, malondialdehyde; SOD, superoxide dismutase.

Other studies demonstrated high levels of hydrogen peroxide and low levels of catalase in the epidermis and peripheral blood of patients with active stages of vitiligo when compared to healthy individuals [67]. Furthermore, oxidative damage to DNA at the level of circulating peripheral blood cells (e.g., lymphocytes and monocytes) was also observed [66]. This once again underlines the involvement, in addition to melanocytes and other skin cells, of multiple actors in vitiligo (Figure 2).

## 4. Mitochondria as Candidates for a Crucial Pathogenetic Role

Mitochondria have recently emerged as potentially involved in vitiligo pathogenesis [68]. In fact, since the mitochondrion is one of the main cellular districts producing ROS, its direct involvement in the pathogenesis of vitiligo is conceivable. Although mitochondria may not seem as indispensable in the skin as in other tissues and organs, such as skeletal and cardiac muscles, kidney, and brain, they are necessary because the skin is a continuously remodeling tissue with regard to pigmentation, wound healing, which also involves the vascular system, as well as hair and hair growth. The most important role played by the skin is that of the first microbial barrier. The skin must therefore be able to respond quickly even to external insults by regulating the necessary energy production from time to time. This makes it particularly dependent on mitochondrial metabolism. Accordingly, some mitochondrial DNA (mtDNA) mutations have been associated with skin anomalies including, but not only, pigmentation [69]. Since the synthesis of melanin is an energetically expensive process and strongly dependent on ATP coming from oxidative phosphorylation (OXPHOS), a defective functioning of vitiligo melanocytes could hyperactivate the mitochondria resulting in increased production of ROS, triggering a redox imbalance [70]. Some researchers have identified transient receptor potential cation channel subfamily M member 2 (TRPM2) and nicotinamide adenine dinucleotide (NAD^+^)-dependent deacetylase sirtuin-3 (SIRT-3) as possible mediators of oxidative damage caused by the altered interaction between pathological melanocyte and mitochondrion [71, 72]. In turn, hydrogen peroxide produced in vitiligo melanocytes has been observed to increase the expression of TRPM2, which, by promoting calcium influx into the mitochondrion, could induce apoptosis of melanocytes [71]. As far as SIRT-3 is concerned, this molecule plays a fundamental role in regulating the mitochondrial fusion process through deacetylation and activation of dynamin-like 120 kDa protein (OPA1) [72], a molecule that can modify the architecture of the mitochondrial cristae and the functionality of the respiratory chain [73]. Hence, it can be hypothesized that the alteration of mitochondrial dynamics, known to activate the apoptotic cascade [74], could play a role in melanocyte integrity and function in vitiligo. Furthermore, both TRPM2 [75] and SIRT-3 [76] can induce autoimmune responses mediated by neutrophils and lymphocytes.

As already mentioned, the process of melanogenesis occurs inside the melanosomes and only subsequently the melanin is transferred to the keratinocytes where it is stored and determines the color of the skin. The transport of melanosomes from melanocytes to keratinocytes is still being studied, but it seems to depend on a tri-protein complex formed by Rab27a, melanophilin, and myosin-Va [77]. This protein complex interacts with prohibitins (1 and 2), pleiotropic proteins that, when localized in the mitochondrion, regulate mitochondrial metabolism and biogenesis [78]. Prohibitins play a dual role in the mitochondria of the skin: by binding to melanogenin, they increase the synthesis of melanin; interacting with the tri-protein complex, they would be involved in the transport of melanin to the melanocytic dendrites from where it is transferred into the keratocytes. It could, therefore, be hypothesized that a reduced interaction of prohibitins with the tri-protein complex mentioned above, e.g., due to a decreased expression of prohibitins or to alterations in the binding sites, could be involved in the pathogenesis or clinical manifestations of vitiligo [79].

**Figure 3:**
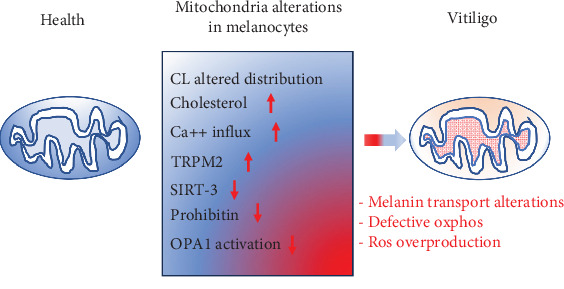
The main mitochondrial alterations found in melanocytes of subjects affected by vitiligo. CL, cardiolipin; OPA1, dynamin-like 120 kDa protein; OXPHOS, oxidative phosphorylation; SIRT-3, nicotinamide adenine dinucleotide (NAD^+^)-dependent deacetylase sirtuin-3; TRPM2, transient receptor potential cation channel subfamily M member 2.

Furthermore, alterations of OXPHOS have been observed in vitiligo melanocytes. In particular, the expression of some electron transport chain proteins and the activity of the complex I were found altered in vitiligo melanocytes [23]. These functional deficits have been hypothesized to be related to alterations in the amount and distribution of cardiolipin (CL) in the membrane of the mitochondria capable of compromising the correct arrangement of the proteins of the respiratory chain. The functional deterioration of the mitochondrion results in reduced ATP production, only partially counteracted by glucose metabolic supply [24]. Therefore, although still to be confirmed, there is growing evidence of a possible crucial role of mitochondria in the pathogenesis of vitiligo (Figure 3).

## 5. The Involvement of Catecholamines and Adrenergic Receptors

The presence of adrenergic receptors (ARs) in the epidermis contributes to the rapid homeostatic responses of keratinocytes and melanocytes following endogenous and exogenous stimuli. Following binding with catecholamines, ARs transduce the signal through second messengers such as cAMP, guanosine-3′,5′-cyclic monophosphate (cGMP), and calcium. It has been observed that the calcium level in the skin determines the density of *β*2-ARs. These receptors in the epidermis are involved in cell growth, motility, and differentiation, as well as pigmentation [80].

In keratinocytes, which express only the *β*2-AR, the increased intracellular calcium concentration inhibits proliferation while increasing differentiation [80]. In addition, stimulation of *β*2-ARs in these cells, through the activation of the protein phosphatase 2A (PP2A), also inhibits their migration, strongly impacting the wound re-epithelialization and the restoration of the epidermal barrier [81].

Keratinocytes have been shown to possess the entire biochemical apparatus for the autocrine synthesis of the catecholamine epinephrine, a selective agonist of *β*2-AR. Epinephrine is synthesized in these cells from the essential amino acid L-phenylalanine [82]. In contrast, melanocytes are considered norepinephrinergic cells. Keratinocytes of the basal layer produce high levels of epinephrine compared to the suprabasal keratinocytes, it has, therefore, been hypothesized that epinephrine, by activating the *β*2-ARs, could induce their differentiation through an increase in intracellular calcium, thus contributing to the correct architecture of the epidermis.

In melanocytes, the activation of the *β*2-ARs stimulates melanogenesis [82]. Thus, keratinocytes through the production of catecholamines can support the synthesis of melanin. Since it has also been observed that systemic beta blockers induce an exacerbation of the disease in some patients, a role in the pathogenesis of vitiligo has been proposed for the catecholamine signaling system, especially mediated by the *β*2-ARs [83]. To corroborate this hypothesis, there is also the observations of an altered metabolism of catecholamines and an anomalous expression of the *β*2-ARs in the skin of subjects affected by vitiligo [83]. In particular, in keratinocytes of vitiligo patients, an increase of the *β*2-ARs expression and a defective extracellular calcium uptake were found. Accordingly, stressful events and the emotional state are considered as important both in the onset and exacerbation of vitiligo; thus, it is plausible that catecholamine-based stress could play a key role.

If further studies could confirm the central role of the catecholaminergic system in the onset and/or progression of vitiligo, the pharmacological modulation of the *β*2-ARs could represent an innovative therapeutic approach to replace or integrate those currently available.

## 6. MicroRNAs in Vitiligo Pathogenesis

MicroRNAs (miRNAs) are small RNA molecules that do not code for proteins, but in their mature form (~23 nucleotides in length) can posttranscriptionally regulate gene expression [84]. They inhibit gene translation by binding to specific sites in the mRNA 3′ untranslated regions (UTR) and, depending on the accuracy of the base pairing, degrade or not their targeted mRNAs [85]. MiRNAs play a crucial role in skin homeostasis mainly during melanocyte differentiation and maturation. Furthermore, deregulated miRNA expression in vitiligo affects the growth, differentiation, and death either in melanocytes or in other cells functionally connected with them. In this review, we will focus on the miRNAs that have a direct or indirect impact on melanocyte biology and whose mRNA targets have been identified. Then, we will discuss in a separate section noncoding RNAs, including circular and long noncoding RNAs as part of the miRNA regulatory network.

### 6.1. MicroRNAs Are Associated With Melanocytic Growth, Differentiation, and Function

The microphthalmia-associated transcription factor (MITF) is the master gene that controls the identity and differentiation of melanocytic cells. MITF regulates numerous genes playing a role in the production of melanin, the pigment responsible for the color of skin, hair, and eyes [86, 87]. Sun exposure induces stress and pigmentation response in melanocytes. In this context, the role of MITF is pivotal, allowing melanocytes to activate protective programs for melanin production to minimize skin damage [88]. Due to this prominent role, several studies have investigated the relationship between miRNAs and MITF in vitiligo pathogenesis. In a study on serum and vitiligo lesions, Shi et al. [89] indicated that high levels of miR-25, derived from demethylation of the miRNA promoter region, occurred in both keratinocytes and melanocytes, ultimately leading to melanocyte degeneration. The expression of miR-25 directly inhibited MITF expression with increased oxidative stress, resulting in melanocyte dysfunction and destruction. Additionally, the impaired protective paracrine program from keratinocytes, where miR-25 activity inhibited SCF and bFGF expression reducing their production and secretion, contributed to melanocyte degeneration [89]. In a study that analyzed the serum and melanocytes of vitiligo patients, the inverse expression of miR-125-5b and MITF has been observed. Functionally, the expression of miR-125b-5p reduced melanocyte proliferation and melanogenesis, downmodulating melanin production-related proteins such as tyrosinase (Tyr) and tyrosinase-related proteins 1 and 2 (TYRP1 and TYRP2). However, MITF overexpression reversed these effects [90].

Microarray analysis on melanocytes with MITF knocked down identified modulated miRNAs possibly involved in the regulation of the expression of the main melanogenic enzymes [91]. In a study conducted by Wang et al. [92], MITF-KO significantly reduced the expression of TYR and TYRP1. In these samples, the reduced expression of melanogenic genes by MITF-KO was associated with upregulation of miR-1225-3p, miR-634, miR-197, miR-766, and miR-574-5p and downregulation of miR-328, miR-720, and miR-1308 [92]. Unfortunately, the majority of these miRNAs are orphans of their putative targets and their possible role in vitiligo pathogenesis waits to be validated. A microRNA signature from murine melanocytes after treatments with forskolin (a known stimulator of the cAMP pathway) and solar-stimulated UV irradiation to induce hyperpigmentation demonstrated the downregulation of the miRNA-145 as a regulator of the pigmentation process by controlling the expression of the melanogenic genes Tyr, Tyrp1, and Myo5a, upregulated by these treatments [93]. The reduced expression of Myo5a might inhibit melanocytic proliferation and promote apoptosis since Myo5a is crucial in transporting melanosomes from melanocytes to keratinocytes. Myo5a might be decreased by miRNA-145 upregulation, a miRNA found upregulated in miRNA expression profiles from active nonsegmental generalized vitiligo samples [94]. This targeting might influence melanosome transfer by blocking their extrusion, impacting melanocyte vitality [95]. This phenomenon is often observed in the hypopigmented skin of patients with Griscelli syndrome, but it is likely to occur in vitiligo [96].

A further epigenetic determinant is represented by MiR-155, which is present in large quantities in the skin of individuals with vitiligo. Its expression leads to the suppression of multiple genes involved in the pigmentation pathway, such as the suppressor of cytokine signaling1 (SOCS1) and TYRP1, ultimately contributing to the development of the pathogenic characteristics of vitiligo [97].

As described above, vitiligo affects various cell types, including keratinocytes and the transfer of miRNAs by exosomes from keratinocytes to melanocytes is crucial for the production of melanin. It is well known that these two types of cells are in close contact, and the biochemical signals received by melanocytes through exosomes are essential for the production and secretion of melanosomes with high melanin content. Then, melanosomes move to keratinocytes to protect them against UV rays and at the same time preserve melanocytes from toxicity derived from melanin accumulation. The dysfunction of keratinocytes and the defective release of exosomes can ultimately affect the function of melanocytes [98]. In this setting, in patients with vitiligo, Zhao et al. [99] found a reduced expression of miR-200c, which weakens the exosome trafficking in melanocytes, thereby affecting the regulation of melanin synthesis. In addition, the reduction of miR-200c results in the misregulation of SOX1 and the block of beta-catenin activity [99]. These two proteins are essential for melanogenesis being positive regulators of MITF expression. Furthermore, for a correct maturation and function of melanin, after the melanosome is taken in by keratinocytes, the expression of TYRP1 is necessary. Unexpectedly, Vaish et al. [100] demonstrated that, beyond melanocytes, this protein is expressed in normal keratinocytes. In fact, in keratinocytes where TYRP1 is downregulated by the action of the miR-202-3p, miR-525-5p, and miR518a-5p, the crosstalk with melanocytes is altered, making the melanosome transfer to keratinocytes defective, an event that triggers melanin accumulation and cytotoxicity in melanocytes [100].

### 6.2. MicroRNAs Associated With Melanocytic Metabolism and Stress in Vitiligo

The stressful environment for the melanocytes and their surroundings in vitiligo can cause inflammation, the creation of new antigens, and exposure to immune system attacks [101]. Prolonged stress conditions reduce enzymes with antioxidant action in parallel to the prolonged oxidation state and accumulation of catecholamine, hydrogen peroxide (H_2_O_2_), and other ROS [102]. Although the molecular basis of melanocyte death associated with the oxidative state remains substantially unclarified, the evidence of crosstalk between miRNA and oxidative stress indicates the presence of a regulatory network capable of mediating the pathogenesis of vitiligo. In this crosstalk, keratinocyte protective role preserves melanocytes from oxidation. Some microRNAs that play a protective role are lost in vitiligo. For instance, the absence of miR-211 is a contributing factor in the development of vitiligo. MiRNA-211 is highly expressed in normal melanocytes and plays a crucial role in the formation of melanin by targeting transforming growth factor beta 2 (TGF*β*2), known for its inhibitory effect on melanocyte differentiation. Loss of miRNA-211 expression triggers a cellular stress response that affects the levels of pigmentation. The result is the increment of peroxisome proliferator-activated receptors gamma variant C1A (PAAR*γ*C1A), ribonucleotide reductase regulatory subunit M2 (RRM2), and thousand-and-one amino acid kinase (TAOK1) protein targets [24, 103]. These proteins cause an abnormal respiratory response in melanocytes, leading to an imbalance in oxidative metabolism and increased production of ROS, negatively affecting melanocyte function and vitality.

MiRNA targeting of stress-responsive elements, antioxidant, and antiinflammatory factors affects melanocyte survival. MiRNA-1 expression induces oxidative stress leading to melanocyte death. This miRNA was previously identified in the targeting of cardiomyocyte heat shock protein-60 and -70 mRNAs and the same mechanism might be functional in vitiligo [104]. MiR-135 and miR-9 can modulate melanocytic cells by targeting SIRT1, a protein that, in response to stress or inflammation, positively regulates cellular processes for melanocyte survival [105]. Additionally, miR-9 has a dual function as it is also present in keratinocytes and is responsible for reducing melanocyte migration. In keratinocytes, miR-9 suppresses the expression of E-cadherin and *β*1 integrin [106]. It is worth noting that ultraviolet radiation B (UVB) exposure for skin repigmentation triggers the production of IL-10, which causes miR-9 to be silenced by methylation ultimately promoting the re-expression of E-cadherin and *β*1 integrin in keratinocytes, restoring the functional migration of melanocytic cells to keratinocytes [106].

A further factor in this very complex scenario is represented by miR-183. This was previously suggested to be involved in the promotion of osteoclast differentiation by direct inhibition of heme oxygenase 1 (HO1), a stress-responsive gene with antioxidant and anti-inflammatory functions [107]. In osteoclast cells, miR-183 expression contributes to bone resorption and osteoporosis. Strikingly, the same regulation occurs in vitiligo patients, where the anti-inflammatory and antiapoptotic functions of the HO1 gene are lost by increased levels of miR-183, an event that negatively affects melanocyte vitality [108].

In normal conditions, miRNA-loaded exosomes can contribute to melanocytic wellness, while, in pathological conditions, altered miRNA expression in keratinocytes may contribute to the disease pathogenesis. High levels of dopamine in vitiligo patients induce ROS levels. This increment negatively influences melanocyte proliferation and melanin synthesis [109, 110]. Recently, it is evidenced that miR-493-3p expression, originated by keratinocytes and loaded in circulating exosomes, was significantly increased, contributing to the disease progression. MiR493-3p targets the ribonucleoprotein U (hnRNPU), which in turn reduces the control on the catechol-*o*-methyltransferase (COMT), a degradative enzyme of dopamine, thus favoring its accumulation [111].

**Figure 4:**
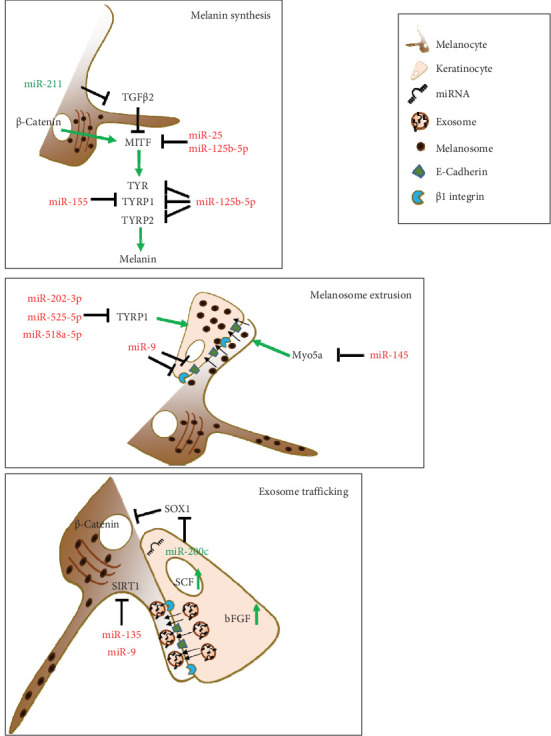
Schematic representation of the main microRNA pathways and their targeted proteins involved in skin physiology, whose dysregulations are associated with vitiligo pathogenesis. In red are reported miRNAs upregulated in vitiligo, while in green those normally expressed in healthy skin and downregulated in vitiligo (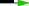
Indicates “induction”, 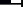 “repression”).

The different contributions of miRNAs in vitiligo pathogenesis have also been evaluated from a sex-based point of view. Mansuri, Singh, and Begum [112] identified a significantly increased expression of miR-383 in female compared to male patients with generalized vitiligo. Although this sex difference has not been deeply investigated, a target predictive analysis pointed at TYRP1 and EDN1 as possible targets of miR-383. EDN1 is a paracrine growth factor that interacts with a-MSH and affects melanocyte proliferation, migration, tyrosinase activity, and melanogenesis. In vitiligo, it can be hypothesized that when miR-383 is deregulated, the miR-dependent downregulation of TYRP and EDN1 supports oxidative stress of melanocytes associated with abnormal or incomplete melanin synthesis, eventually favoring the final apoptosis [112]. More relevant miRs playing roles in vitiligo are reported in Table 1. A schematic representation of the main microRNA pathways and their targeted proteins involved in vitiligo pathogenesis are illustrated in Figure 4.

## 7. The Role of Long Noncoding and Circular RNAs in Vitiligo

Two other classes of regulatory RNA, long noncoding RNAs (lncRNAs) and circular RNAs (circRNAs) have recently been indicated to play a role in vitiligo pathogenesis. LncRNAs are widely expressed noncoding RNAs more than 200 base pairs in length. They can interact with specific miRNAs, reducing their stability, and acting as sponges or molecular baits of miRNAs [113], competing with miRNAs for target mRNA [114], and generating miRNAs to silence the target mRNA [115].

On the other hand, circRNAs are stable, abundant noncoding RNAs with covalently closed-loop structures. They act as miRNA sponges by binding miRNAs with miRNA-responsive elements (MREs), thus influencing gene expression [116]. Several studies have demonstrated abnormal expression of lncRNAs and circRNAs in various human diseases, indicating that they may become new markers for disease detection [117]. Zhang et al. [118] using high-throughput RNA sequencing, to reveal differentially expressed RNAs, explored the patterns of expression for lncRNAs in peripheral blood mononuclear cells (PBMCs) from patients with advanced nonsegmental vitiligo and healthy individuals. Results, validated by qRT-PCR, demonstrated two potential differentially expressed lncRNA: ENST00000460164.1 and NR-046211.1. The lncRNA-miRNA-mRNA network prediction analysis individuated 17 miRNAs that may bind to these lncRNAs. Among them, via bioinformatic methods, the ENST0000460164.1 lncRNA was indicated as the sponge of mir-637, predicted to target IL16 and IL34; the NR-046211.1 lncRNA was predicted to bind with mir-328 generally downmodulated in skin lesions and found to regulate the oxidative stress mechanism by targeting IL1b. Therefore, although preliminary, this study indicates ENST00000460164.1 and NR-046211.1 as the two potential biomarkers and drug targets for the treatment of nonsegmental vitiligo [118]. Significant results have been obtained from research conducted by various groups, which identified the lncRNA metastasis-associated lung adenocarcinoma transcript 1 (MALAT1) as being involved in protecting the amelanotic keratinocytes of vitiligo patients from UV radiation [119]. The increase in MALAT1 expression leads to a decrease in miR-211 expression. MALAT1 specifically binds to and suppresses miR-211, increasing the expression of the miR-211 target SIRT1. SIRT1 is a crucial protein for keratinocyte differentiation and protection from UV-induced DNA damage. These findings establish a new MALAT1–miR-211–SIRT1 signaling pathway that may provide long-term protection to the keratinocytes of the amelanotic skin of vitiligo patients, potentially explaining their lower risk of developing melanoma and non-melanoma skin cancer [120]. In vitiligo patients, the expression of the lncRNA Taurine-upregulated gene 1 (TUG1) was found to be decreased compared to healthy controls. Additionally, PPAR-*γ* levels were significantly reduced in association with lncRNA TUG1. Conversely, miRNA-377 and IL-17 were significantly upregulated in the vitiligo group compared with the control group, indicating a potential involvement in disease pathogenesis through PPAR-*γ* downregulation and IL-17 and miR-377 upregulation [121].

A second group of regulatory RNAs known as circRNAs has recently been studied concerning vitiligo pathogenesis. Researchers analyzed differentially expressed circRNAs using microarray analysis of skin biopsies from vitiligo patients and compared them to healthy control skin. The study revealed two differentially expressed circRNAs: hsa_circRNA_000957 and hsa_circRNA_101798, upregulated and downregulated, respectively. These circRNAs contained 34 MREs associated with 162 target genes potentially involved in vitiligo [122]. In a more specific study, researchers identified a circ_0087961-miR27a-3p-Paxillin network that might play a role in vitiligo. Paxillin, a protein that promotes the adhesion ability of melanocytes, is lost in vitiligo due to the upregulation of miR27a-3p in association with the downmodulation of the circ_0087961 RNA [123].

Scientific evidence enlightens connections between abnormal noncoding RNA expression and human disorders (Table 2). In vitiligo, advances in RNA-sequencing methods have led to the discovery of numerous noncoding RNAs with unknown functions that, if elucidated, will provide specific information about their role in a physiological or pathological context. However, further studies are necessary to understand how these RNAs are involved in vitiligo pathogenesis to confirm their specific association as disease biomarkers or novel therapeutic targets for future treatment.

## 8. Metabolic Signature in Vitiligo

Cellular metabolic alterations resulting in changes in the lipid composition of cell membranes in subjects affected by active nonsegmental vitiligo have already been reported in the past. In particular, the reduced expression of CL and the increase in cholesterol content had been associated with an altered arrangement of transmembrane proteins in the mitochondrion with consequent deterioration in the functional mitochondrial parameters. Vitiligo could therefore be considered a mitochondrial pathology characterized by an intracellular redox imbalance, strictly related to the loss of efficiency of the mitochondria, in which glucose and lipid metabolism are compromised [23].

In recent years, metabolomic studies conducted with different methodologies have highlighted a clear separation between patients with vitiligo and healthy controls in terms of lipid metabolism [124, 125]. Metabolomics provides valuable qualitative and quantitative information on low-molecular-weight metabolites that reflect the pathophysiological state of an individual and are potentially important in pathogenesis, as diagnostic or prognostic biomarkers and/or potential therapeutic targets.

More than one study highlighted the association between lipid metabolism alteration and vitiligo, both at systemic and cellular level [126, 127]. A very recent analysis conducted on a limited number of subjects (50 patients with nonsegmental vitiligo and 50 matched control subjects) found a different lipidic profile with higher levels of total cholesterol (TC), LDL, and triglycerides (TG), but lower levels of high-density lipoprotein (HDL) in vitiligo patients. Significantly increased expression of circadian genes was also reported in patients with nonsegmental vitiligo, with increased detection of Basic Helix-Loop-Helix ARNT Like 1 (BMAL1) and its polymorphic variants (single-nucleotide polymorphismn T/C *vs* T/T genotypes) [128], whose product is a clock transcription factor protein that generates circadian rhythms in physiological functions. Interestingly, stimulation of BMAL1 by environmental ligands is implicated in the regulation of glucose and lipid metabolism, and its dysregulation has been linked to metabolic syndrome [129]. Therefore, the authors proposed the circadian gene as a useful marker for early diagnosis, although it cannot be proposed to determine the severity of vitiligo.

A metabolomic study conducted on 48 vitiligo patients (24 with active vitiligo, 24 with stable vitiligo) and 28 healthy individuals provided evidence that vitiligo was associated with dysregulated polyunsaturated fatty acid metabolism. The authors found that serum level of alpha-linolenic acid (ALA) was significantly upregulated, while that of arachidonic acid (ARA), arachidic acid (AA), and behenic acid was significantly downregulated in vitiligo patients. Furthermore, their analyses also suggested that the concentrations of ALA could partly reflect the severity of vitiligo [130].

Other studies have also shown an alteration of the Sphingosine-1-phosphate (S1P) plasma levels in subjects with vitiligo compared to healthy donors. Furthermore, it has been hypothesized that the high level of S1P in healthy people could represent a risk factor in the pathogenesis of vitiligo [131]. S1P is a signaling sphingolipid, also known as lysosphingolipid S1P, that may inhibit melanin production from melanocytes [132], and is involved in the recruitment of immune cells during inflammation [133]. Two additional elements suggest that S1P is involved in the pathogenesis of vitiligo: (1) the blockade of recirculating memory T cells recruitment to the skin by administration of FTY720, an S1P receptor modulator, is able to induce repigmentation in a mouse model of vitiligo [134]; (2) the sphingolipid signaling was enriched only in vitiligo melanocytes but not in normal human melanocytes [135]. This led to the hypothesis of S1P as a potential therapeutic target for vitiligo.

Since pigment metabolism is known to play a significant role in the pathogenesis of vitiligo, Marzabani and coworkers explored amino acid metabolism in vitiligo using targeted metabolomics on 31 patients with nonsegmental vitiligo, and 34 healthy individuals. Eight amino acids, namely cysteine, arginine, lysine, ornithine, proline, glutamic acid, histidine and glycine were observed to be differentially detectable in vitiligo subjects compared to healthy controls. Specifically, cysteine, glutamic acid and proline increased, while arginine, lysine, ornithine, glycine and histidine decreased significantly in vitiligo patients compared to healthy individuals. The authors also found that glutamic acid was significantly increased in patients with less than 25% involvement, compared to the others, in terms of affected skin area. Interestingly, the analyses suggested cysteine and lysine as promising candidates for the diagnosis and risk of vitiligo development [125].

Relatively recent studies have highlighted the presence of metabolic comorbidities in subjects with vitiligo, clearly showing as vitiligo is associated with an unfavorable lipid profile and could therefore represent a cardiovascular risk factor [136]. It is conceivable that the systemic proinflammatory state associated with vitiligo may also contribute to cardiovascular increased risk by favoring atherosclerotic phenomena in patients. These studies, published between 2020 and 2024, are unanimous in reporting a significantly higher incidence of metabolic syndrome in patients with severe vitiligo (higher Vitiligo Area Severity Index). A positive correlation between the syndrome and the activity and duration of vitiligo was also reported in these studies [137, 138]. In addition, significantly higher values of fasting glucose, low-density lipoprotein (LDL) cholesterol and blood pressure were observed compared to the control group. By contrast, HDL levels recorded were generally lower compared to healthy controls [139]. Considering the significantly higher prevalence of metabolic syndrome in vitiligo patients, especially in those with active disease, constant monitoring of vitiligo patients could promote early diagnosis of metabolic syndrome, reducing the risk of cardiovascular disease in this population.

## 9. Sex Hormones and Vitiligo

The multifactorial origin of vitiligo etiopathogenesis makes it difficult for researchers to determine the contribution of sex hormones to this skin disease.

In addition to sexual development and reproduction, it is widely recognized that sex hormones influence the immune response, with estrogens acting as activators while androgens and progesterone as suppressors of the immune system. Additionally, sex hormones play a significant role in skin homeostasis, and extensive literature describes receptors for estrogen, androgen, progesterone, and prolactin (PRL) on various cells of the epidermis and dermis. Numerous epidemiological and clinical studies have observed sex differences in numerous skin pathologies. However, all efforts made so far to determine the potential role of sexual hormones in the etiology of vitiligo have not achieved robust and incontrovertible results. Therefore, in this paragraph, we will provide an overview of the data published on sexual hormones and vitiligo, without neglecting the contradictions present in the literature.

Based on epidemiological studies, no significant sex differences have been reported on vitiligo onset and progression in adult patients, except for the most affected body sites [140]. Furthermore, males more frequently reported a family history of vitiligo and a longer duration of disease compared to females [14]. Nevertheless, the ratio of treatment requests from females to males is ~2:1, indicating that women are more sensitive to cosmetic disorders and seek treatment earlier [141]. Conversely, a slight prevalence of vitiligo among females has been observed in childhood, both in pre- and post-pubertal phases compared to males [142]. It cannot be ruled out, however, that even younger girls are more likely to seek medical advice than boys.

On the contrary, Khurrum and AlGhamdi [143] reported more males in the postpubertal group than females, who were more represented in the prepubertal group showing face involvement. Differences in analysis methodologies, sample size, age, and skin color along with many other variables are likely responsable of most contradictory results in the literature.

In the past, the association of pregnancy or contraceptive use with altered cutaneous pigmentation, notably melasma, an epidermal hyperpigmentation affecting sun-exposed areas of the skin, was proposed. Indeed, it has been determined that sex hormones, along with other key factors, such as UV exposition, patient age, and family history, may contribute to melasma development [144]. With regard to pregnancy, recent studies have highlighted that the vitiligo disease remains mostly stable, with few cases of improvement and rare cases of worsening [145, 146].

Besides epidemiological analysis, several studies aimed at investigating the levels of the main sex hormones in patients' serum (Table 3). With regard to estrogen, some researchers have provided evidence supporting a direct hormonal action on skin cells, especially melanocytes. Interestingly, increased levels of estrogen have been detected in the serum of female patients within the active vitiligo group (AVP) [147] or more generally in female patients compared to healthy ones [148]. Previously, Sabek et al. [149] also demonstrated a decrease in estrogen receptor (ER) *β* expression in female patients.

The biological effects exerted by 17*β*-estradiol on cultures of human epidermal melanocytes were analyzed, and the presence of functionally active ERs in melanocytes was demonstrated [153, 154]. Melanocytes were found to respond to estrogenic stimuli increasing their proliferation and tyrosinase activity, leading to higher melanin content and its extrusion [154]. Conversely, conflicting findings were reported in other studies, partly due to differences in culture conditions and the melanocyte donor used [154, 155]. However, more recently, Natale et al. [156] demonstrated that epidermal melanocytes express a membrane-associate ER: the steroid hormone receptor G protein-coupled estrogen receptor (GPER), which, localized at the plasma membrane, was able to induce very rapid effects in cells and, as concern melanocytes, pigment production.

It is widely known that, in addition to the ovaries, estrogens are synthesized by other tissues, mainly adipose and cutaneous. Indeed, the skin is considered the largest peripheral endocrine organ, where different cell populations, acting in a coordinated way, can produce or metabolize different types of hormones, including sex hormones and PRL. These hormones can function locally, via paracrine/autocrine pathways, and systemically throughout the entire organism [157]. In this regard, a recent paper [158] has demonstrated that the 17*β*-hydroxysteroid dehydrogenase 1 (HSD17*β*1) was the most abundant enzyme of estrogen synthesis localized in keratinocytes. Furthermore, through the analysis of several cultured human skin cells, the authors showed that estrogen receptors were mainly present in melanocytes, with GPER 1 being the most expressed, thus confirming the findings of Natale et al. [156]. The function of locally synthesized estrogen has been shown to protect melanocytes from oxidative stress. In line with this result, decreased levels of HSD17*β*1 were found in skin lesions from vitiligo patients compared to samples from healthy donors [158]. Conversely, previous studies have shown that estrogenic stimulus led to the generation of hydrogen peroxide which, in turn, caused DNA damage in peripheral blood lymphocytes of vitiligo patients [159]. It is, however, well known that the concentration of estrogen could be critical, capable of exerting antioxidant activities at low doses and prooxidant effects at higher concentrations. Thus, the skin could gain great relevance regarding the production of estrogen, considering the hormonal differences that occur in pre- and post-menopausal women.

Progesterone is another important female sex hormone extensively studied in vitiligo. It has been demonstrated to counteract estrogen activity [160] and to reduce pigment production in human melanocytes through inhibitory subunits of the noncanonical G protein-coupled receptor PAQR7 (progestin and adipoQ receptor 7) [156]. It is worth noting that this work, in addition to the well-known MC1R, identified other GPCRs, namely GPER1 and PAQR7. All these receptors are capable of directly influencing melanin production through the common cAMP signaling pathway upon specific stimulus, thus suggesting a finely regulated balance between the activities carried out by estrogen, progesterone and *α* melanocyte-stimulating hormone (*α*MSH), a 13 amino acid peptide with potent anti-inflammatory effects.

Androgens are also known to exert a significant impact on the skin cells. The skin can metabolize androgens into dihydrotestosterone (DHT), and androgen receptors are present in various cutaneous cells [161]. An *in vitro* study on normal human melanocytes demonstrated that testosterone and DHT were able to reduce tyrosinase activity and intracellular cAMP levels [162]. Regarding testosterone levels in the serum of patients (Table 3), a significant decrease was observed in male patients compared to healthy ones, with the lowest level in the active vitiligo group [147]. Conversely, Hussein et al. [148] showed an increase of testosterone in male patients' serum but not in females compared to control.

In this hormonal overview we cannot but mention the PRL, a polypeptide hormone responsible for lactation and reproduction, exerting various physiological functions including the maintenance of skin homeostasis [163]. Although not considered a classical sexual hormone, prolactin has received much attention from researchers to uncover a sex-linked etiology of vitiligo. Both prolactin and its receptor (prolactin receptor, PRLR) are expressed in several cutaneous cell populations. Furthermore, PRL is an important modulator of cellular and humoral immunity [164].

Consistently, PRL levels have often been associated with various autoimmune diseases, including psoriasis [165]. With regard to for the studies on patients with vitiligo, contradictory results have been published so far about the presence of significantly increased serum prolactin levels compared to healthy subjects: some authors have found increased levels [147, 150], whereas others do not [151, 152] (Table 3). In any case, no differences between sexes were observed. The positive correlation observed between tissue prolactin and PRLR levels in vitiligo patients, supported the hypothesis of an autocrine/paracrine activity of prolactin able to modify the melanocyte microenvironment [150]. However, it is worth mentioning that PRL is also able to modulate stress response and regulate emotions [166] so that, from this point of view, prolactin could influence vitiligo at the psychological level.

In conclusion, sexual hormones exert different functions, locally in the skin through autocrine/paracrine mechanisms and throughout the body via peripheral blood circulation. Furthermore, their actions are interconnected with each other and with a complex network of neuroendocrine and immunostimulatory signals that collectively regulate various aspects of skin physiology. Changes in this fine balance of signals can lead to or be associated with vitiligo.

## 10. A Further Look at Sex and Gender Differences

Beyond the possible role of hormones in vitiligo onset or progression as stated above, no significant sex differences have been reported so far as therapeutic response or disease severity is concerned [167]. However, some points should be considered before this issue could be excluded by the interest of physicians and researchers. In particular, as concern the formers, a couple of works seem to suggest that concomitant pathological conditions could be relevant. For instance, fibromyalgia syndrome, which is prevalent in vitiligo patients compared to controls, shows a significant association with female sex [168]. A further example: male patients with T-cell-mediated autoimmune granulomatous uveitis seem to be at higher risk of vitiligo and worse prognosis than female patients [169]. As concerns basic science studies, a recent work underlines the fact that the full pigmentation recovery is rarely achieved due to the poor understanding of the molecular mechanisms governing this process. The authors suggested that a sexually dimorphic cutaneous inflammatory response generated by ultraviolet B exposure could lead, at least in mice, to different migration rates between melanocyte stem cells from males and females [170]. By contrast, studies carried out on autologous cultured melanocytes transplantation (or skin graft) did not show any significant sex-specific response [171].

However, in line with our previous study [17], the quality of life (QOL) impairment in women (and not in men) affected with vitiligo appears as of major relevance. It equals the impairment caused by psoriasis in a study population. These results should awake the interest of physicians in this “cosmetic” disease, since appropriate treatment is likely to improve the quality of life of patients with vitiligo [172]. In fact, some authors found that women with vitiligo experience greater QOL impairment than their male counterparts and that the overall well-being of such patients is important in their management and policymaking [173]. Despite advances in therapies, vitiligo continues to be difficult to treat and it often presents unwanted effects, especially in individuals with skin of color, in which skin changes are more visible. Various camouflage methods are normally used to conceal the depigmented areas: waterproof makeup, sunless self-tanners containing dihydroxyacetone, professional spray tans, and medical tattoos. It has been observed that cosmetic products are generally marketed toward women and that males were less likely to utilize camouflage than females. It could thus be relevant that dermatologists could discuss camouflage options with all patients including male patients [174].

## 11. Conclusion

From the analysis of the numerous works published over the years on vitiligo, a picture of great biological complexity clearly emerges in which the central role played by the immuno-neuro-endocrine axis appears as highlighted. The autoimmune nature of this pathology has been widely shown by a myriad of studies demonstrating an involvement of both humoral and cellular immunity, as well as the constant association between disease activity and the presence of both local and systemic inflammation [175]. The role of the nervous system, already hypothesized in the 1950s [176], seems to be suggested by the distribution of the lesions often observed in vitiligo [177]. In fact, the most commonly involved dermatome (areas of the skin whose sensory distribution is innervated by the afferent nerve fibers from the dorsal root) was the trigeminal, whereas only a few patients had an associated autoimmune disease [178]. Furthermore, the sympathetic nervous system, through catecholamines and its peripheral receptors, together with the neurotransmitter serotonin, participates in skin pigmentation and has been observed to be involved in the development of vitiligo [147]. With regard to the endocrine aspect, adrenocorticotropic hormone, melanocyte-stimulating hormone, estrogen, and progesterone stimulate the synthesis of melanin. In particular, estrogens act through the stimulation of tyrosinase activity in melanocytes [146], so that the use of estrogens and progesterone in the prevention of vitiligo was hypothesized [154]. In fact, tyrosinase activity is very important: if uncontrolled it can result in increased melanin synthesis so that decreasing tyrosinase activity has been targeted for the improvement or prevention of conditions related to the hyperpigmentation of the skin, including melasma and age spots. Conversely, increased testosterone levels have been associated with decreased pigmentation. In fact, testosterone, in addition to blocking the activity of tyrosinase [179], would also decrease the expression of the melanocortin 1 receptor [180], one of the key proteins involved in regulating mammalian skin color.

Nonetheless, literature data underline how local and systemic oxidative stress represents both one of the main pathogenetic factors and the hallmark of this disease.

Unfortunately, despite the growing data on the biological aspects of the disease and the deeper knowledge of the biochemical and molecular pathways involved in vitiligo onset and progression, no effective therapy has been identified so far that could guarantee a satisfactory response to all patients. A further obstacle is represented by the lack of one or more biomarkers that can give indication on the state of the disease and on the biochemical pathways most involved in each clinical case that can help clinicians in choosing the most promising individual therapeutic option. In consideration of the complexity of the disease, it is therefore desirable to arrive at a therapy design based on the biological aspects most involved and the specific characteristics of each patient in the current perspective of an increasingly personalized medicine.

## Figures and Tables

**Table 1:** Most relevant MiRs playing a role in vitiligo.

Functions in vitiligo	MiRNA expression	Regulation	Source	Target gene	Methodology	Refs.
Melanocyte function	miR125-b5p	Upregulated	Serum and lesional skin tissues	MITF	qRT-PCR	[90]
	miR-1225-3p, miR-634, miR-197, miR-766, miR-574-5p, and miR-328	Upregulated	MITF-KO melanocytes	MITF	Microarray study	[92]
	miR-720 and miR-1308	Downregulated	MITF-KO melanocytes	MITF	Microarray study	[92]
	miR-145	Downregulated	Forskolin and ssUV stimulation in murine melanocytes	Mitf, Tyr, Trp1, and Myo5a	qRT-PCR	[93]
	miR-155	Upregulated	Lesional skin tissues	SOCS1 and TRP1	In situ hybridization and qRT-PCR	[97]

Keratinocytes/melanocytes crosstalk	miR25	Upregulated	Serum and lesional skin tissues	bFGF and SCF	Microarray study	[89]
	miR-200c	Downregulated	Isolated exosomes from keratinocytes of vitiligo lesions	SOX1	qRT-PCR	[99]
	miR-202-3p, miR-525-5p, and miR518a-5p	Upregulated	Lesional skin tissues	TRP1	Microarray study	[100]
	miR-9	Upregulated	Lesional skin tissues and HaCaT cell line	E-cadherin and *β*1 integrin	qRT-PCR	[106]

Oxidative stress affecting melanocytic function and vitality	miR25	Upregulated	Serum and lesional skin tissues	MITF	Microarray study	[89]
	miR-211	Downregulated	Lesional skin tissues and human vitiligo cell line PIG3V	TGFβ2, PAARγC1A, RRM2, and TAOK1	qRT-PCR	[103]
	miR-1	Upregulated	Lesional skin tissues	HSP60, HSP70	Low-density array	[104]
	miR-135a and miR-9	Upregulated	Lesional skin tissues	SIRT1	Low-density array	[105]
	miR-183	Upregulated	Lesional skin tissues	HO1	Low-density array	[108]
	miR-493-3p	Upregulated	Circulating exosomes from patients	hnRNPU	Microarray study	[111]
	miR-383	Upregulated in female	PBMC from patients with vitiligo	TRP1 and EDN1	Low-density array	[112]

*Note:* The functions they regulate, the target genes, the affected cytotype, and the source are specified.

Abbreviations: bFGF, basic fibroblastic growth factor; MITF, microphthalmia-associated transcription factor; SCF, stem cell factor; SOCS1, suppressor of cytokine signaling1.

**Table 2:** Most relevant lncRNAs and circRNAs with a role in vitiligo pathogenesis.

Function in vitiligo	lnc/circRNA	Regulation	Source	Regulated miRNA	Regulated miRNA-target	Refs.
Inflammation	lncRNA ENST00000460164.1	Upregulated	PBMCs	miRNA-637	IL16, IL34	[118]
Oxidative stress	lncRNA NR-046211.1	—	PBMCs	miRNA-328	IL1b	[118]
Protection from UV-induced DNA damage	lncRNA MALAT1	Upregulated	Keratinocytes	miRNA-211	SIRT1	[120]
Oxidative stress and melanocyte disfunction	lncRNA TUG1	Downregulated	Serum	miRNA-377	PPAR-*γ*	[121]
Melanocyte adhesion	hsa_circ_0087961	Downregulated	Melanocytes	miR27a-3p	Paxillin	[123]

*Note:* In this table, only the lncRNAs and circRNAs for which miRNAs and the related gene target have been identified are specified, along with their possible functions and cell source.

Abbreviations: circRNAs, circular RNAs; lncRNAs, long noncoding RNAs; miRNAs, microRNAs; MALAT1, metastasis-associated lung adenocarcinoma transcript 1; PBMCs, peripheral blood mononuclear cells; TUG1, taurine-upregulated gene 1.

**Table 3:** Estrogen, testosterone, and prolactin levels in the serum of vitiligo patients compared to healthy volunteers.

Serum hormone	VP vs. HV (female)	VP vs. HV (male)	VP vs. HV (total)	Sample size	Refs.
Estrogen	Significantly increased (AVP)	Not significant differences	Significantly increased (AVP)	60 VP vs. 40 HV	[147]
	Significantly increased	Not significant differences	n.d.	30 VP vs. 30 HV	[148]
	Significantly increased	Significantly increased	Significantly increased	60 VP vs. 60 HV	[149]

Testosterone	Not significant differences	Significantly decreased (AVP and SVP)	Decreased (AVP and SVP)	60 VP vs. 40 HV	[147]
	Not significant differences	Significantly increased	n.d.	30 VP vs. 30 HV	[148]

Prolactin	Not significant increase	Not significant increase	Significantly increased (AVP and SVP)	60 VP vs. 40 HV	[147]
	n.d.	n.d.	Significantly increased (also tissue)	20 VP vs. 20 HV	[150]
	Not significant differences	Not significant differences	Not significant differences	36 VP vs. 40 HV	[151]
	n.d.	n.d.	Not significant differences	40 VP vs. 40 HV	[152]

*Note:* Where described, results are reported for sex-divided group, and the sample size of the study is indicated.

Abbreviations: AVP, active vitiligo group; HV, healthy volunteer; n.d., not described; VP, vitiligo patient.

## Data Availability

The authors have nothing to report.
